# Characterization of Arginase Expression in Glioma-Associated Microglia and Macrophages

**DOI:** 10.1371/journal.pone.0165118

**Published:** 2016-12-09

**Authors:** Ian Zhang, Darya Alizadeh, Junling Liang, Leying Zhang, Hang Gao, Yanyan Song, Hui Ren, Mao Ouyang, Xiwei Wu, Massimo D’Apuzzo, Behnam Badie

**Affiliations:** 1 Division of Neurosurgery, City of Hope Beckman Research Institute, Duarte, California, United States of America; 2 Research Center of Siyuan Natural Pharmacy and Biotoxicology, College of Life Sciences, Zhejiang University, Hangzhou, P. R. China; 3 Department of Bone and Joint Surgery, No.1 Hospital of Jilin University, Changchun, Jilin Province, P. R. China; 4 Department of Nephrology, The Second Hospital of Jilin University, Changchun, Jilin Province, P. R. China; 5 Department of General Surgery, The Second Hospital of Jilin University, Changchun, Jilin Province, P. R. China; 6 Department of Cardiology, Third Xiangya Hospital, Central South University, Changsha Hunan, P. R. China; 7 Department of Molecular and Cellular Biology, City of Hope Beckman Research Institute, Duarte, California, United States of America; 8 Department of Pathology, City of Hope Beckman Research Institute, Duarte, California, United States of America; University of Michigan Medical School, UNITED STATES

## Abstract

Microglia (MG) and macrophages (MPs) represent a significant component of the inflammatory response to gliomas. When activated, MG/MP release a variety of pro-inflammatory cytokines, however, they also secrete anti-inflammatory factors that limit their cytotoxic function. The balance between pro and anti-inflammatory functions dictates their antitumor activity. To evaluate potential variations in MG and MP function in gliomas, we isolated these cells (and other Gr1^+^ cells) from intracranial GL261 murine gliomas by FACS and evaluated their gene expression profiles by microarray analysis. As expected, arginase 1 (Arg1, M2 marker) was highly expressed by tumor-associated Gr1^+^, MG and MP. However, in contrast to MP and Gr1^+^ cells that expressed Arg1 shortly after tumor trafficking, Arg1 expression in MG was delayed and occurred in larger tumors. Interestingly, depletion of MPs in tumors did not prevent MG polarization, suggesting direct influence of tumor-specific factors on MG Arg1 upregulation. Finally, Arg1 expression was confirmed in human GBM samples, but most Arg1^+^ cells were neutrophils and not MPs. These findings confirm variations in tumor MG and MP polarization states and its dependency on tumor microenvironmental factors.

## Introduction

Tumor-associated stromal cells like endothelial, mesenchymal and infiltrating inflammatory cells play an important role in tumor pathogenesis. Among these, myeloid-derived cells are abundant in tumors and have been shown to promote tumorigenesis, angiogenesis and invasion [1]. A class of these cells, designated as myeloid-derived suppressor cells (MDSC), possess immunosuppressive properties that facilitate immune escape based on local microenvironmental factors [2]. MDSCs, however, do not represent a single cell population, but are composed of immature myeloid cells at different stages of cell differentiation. These cells can suppress the immune response by several mechanisms, including the production of arginase 1 (Arg1), which decreases the level of L-arginine that is critical for normal T cell function. Lower levels of arginine are known to reduce T cell receptor chain expression and to promote T cell dysfunction. These cells also secrete nitric oxide and reactive oxygen species which are capable of inducing T cell suppression [3].

In gliomas, myeloid-derived cells are mostly represented by resident microglia (MG) that migrate into the brain during early development, or by infiltrating tumor macrophages (MPs) that arise from circulating monocytes. Although other myeloid cells such as neutrophils and other granulocytes are also present in gliomas, infiltrating MG and MPs (referred to as tumor-associated macrophages or TAMs) have received recent attention due to their involvement in glioma escape from anti-angiogenic agents [4]. As components of the innate immune system, TAMs express a variety of factors that constantly alter tumor microenvironment. These cells can produce proinflammatory molecules such as TNFα, IL1β, and CXCL10 that can both activate antitumor immune responses and support tumor angiogenesis and invasion [5–10]. TAMs may also secret immunosuppressive cytokines like IL-10 and TGFβ and matrix-degrading enzymes like MMP2, MMP9, MT1-MMP and cathepsins that promote glioma invasion, immune escape and angiogenesis. So far, most TAM characterization studies have grouped glioma MG and MP as a single cell population, and the contribution of each cell type to glioma microenvironment has been more difficult to evaluate due to overlapping phenotypic and functional similarities.

In this study, to evaluate potential variations in MG and MPs function in gliomas, we isolated these cells (and other MDSC) from GL261 murine gliomas based on flow cytometry staining characteristics [11]. A genome-wide microarray expression analysis demonstrated significant upregulation of Arg1 in both tumor MG and MPs as compared to circulating monocytes. These studies also suggested significant similarities in gene expression profiles between tumor MG and MP. In contrast to MPs, however, Arg1 expression in resident MG was delayed and occurred later during tumor growth and was independent of TAM infiltration into gliomas. Evaluation of human tumor specimen also confirmed Arg1 expression in both TAMs and other myeloid-derived cells such as neutrophils. These findings confirm dynamic changes in TAM polarization that is dependent on tumor microenvironmental factors and highlights variations in the contribution of MDSCs to the immunosuppressive glioma milleu.

## Materials and Methods

### Reagents and cell lines

Luciferase-expressing GL261 glioma cells (GL261-Luc) were obtained from Dr. Karen Aboody's laboratory in 2006 and were generated as described before [12]. Luciferase-expressing KR158B cells (or K-Luc), an invasive glioma cell line that was derived from spontaneous gliomas in *Trp53/Nf1* double-mutant mice in Dr. Tyler Jacks laboratory, was a generous gift from Dr. John Sampson in 2011 [13]. Both GL261-Luc and K-Luc cells were cultured in DMEM medium supplemented with 10% FBS (BioWhittaker, Walkersville, MD), 100 U/mL penicillin-G, 100 μg/mL streptomycin and 0.01 M Hepes buffer (Life Technologies, Gaithersburg, MD) in a humidified 5% CO2 atmosphere, and their tumorigenicity was authenticated by histological characterization of intracranial gliomas in syngeneic C57BL/6J mice.

### Tumor implantation

Mice were housed and handled in accordance to the guidelines and approval of City of Hope Institutional Animal Care and Use Committee under pathogen-free conditions. All mice were on C57BL/6J background. *CX*_*3*_*CR*_*1*_^*GFP*^ Knock-in mice that express EGFP under control of the endogenous Cx3cr1 locus were purchased from Jackson Laboratory (Sacramento, CA). CD11b-TK^mt-30^ mice, a generous gift from Dr. JP Julien, were bred at our institution and PCR genotyped by using Genotyping DNA preparation Kit (Bioland Scientific LLC). Intracranial tumor implantation was performed stereotactically as described before [14]. Briefly, GL261-Luc or K-Luc glioma cells were harvested by trypsinization, counted, and resuspended in culture medium. Female C57BL/6 mice (Jackson Laboratory, Bar Harbor, ME) weighing 15–25 g were anesthetized by intraperitoneal administration of ketamine (132 mg/kg) and xylazine (8.8 mg/kg) and implanted with 10^5^ tumor cells using a stereotactic head frame at a depth of 3 mm through a bur hole placed 2 mm lateral and 0.5 mm anterior to the bregma.

All mice were housed and handled in accordance to the approved guidelines of City of Hope Institutional Animal Care and Use Committee under pathogen-free conditions.

During experiments, animals were monitored daily. Animals displaying excessive pain or discomfort were given appropriate analgesic treatment (Buprenex 0.05–0.1mg/kg SQ/ approximately 0.13–0.2mL) if necessary. For the survival experiments, animals that exhibited mild to moderate signs of distress (hunched, ruffled, decrease in activity, and body weight loss up to 20%) were monitored closely (at least daily) for up to 3 consecutive days to determine if the signs were transient and due to the inflammatory response or chemotherapy treatment. If at 3 days, the mice are still exhibiting one of the mentioned signs, they were euthanized by CO2. If at any time animals had a seizure, could not reach food or water (due to impaired ambulation, for example), were unable to remain upright, or lost greater than 25% body weight, they were euthanized immediately.

### Flow cytometry analysis and FACS sorting

Tumors, spleen and blood samples from mice were harvested and examined by flow cytometry as described previously [15]. Briefly, tissue was minced, digested with trypsin (20 minutes at 37°C) and filtered through a 40 μm filter and prepared using Fixation/Permeabilization solution according to the manufacturer’s instructions (BD Pharmingen. San Diego, CA). For *ex vivo* staining, tumor Multiple-color FACS analyses was performed at City of Hope FACS facility using a 3-laser CyAn immunocytometry system (Dako Cytomation, Fort Collins, CO) and data was analyzed using FlowJo software (TreeStar, San Carlos, CA) as described before [15]. PerCP-conjugated anti-mouse CD11b (Cat: 557235) was purchased from BD Pharmingen (San Jose, CA). APC-conjugated anti-mouse CD11b (Cat: 17-0012-82) were purchased from eBioscience (San Diego, CA). The primary rabbit-anti-mouse Arginase I was purchased from Thermo Scientific (Waltham, MA). Secondary goat anti-rabbit-FITC (Cat: sc-2012) and secondary goat anti-mouse-FITC (Cat: sc-2010) were purchased from Santa Cruz Biotechnology (Santa Cruz, CA).

For human studies, tumor and blood samples from patients undergoing craniotomy were collected under an Institutional Review Board (IRB)-approved protocol (IRB 07074). Written consent for tissue collection was obtained from every patient. Samples were analyzed by FACS on the day of surgery following Percoll isolation.

### Microarray and bioinformatic analysis

FACS sorted cells were collected and total RNA was isolated using TRIzol (Invitrogen). The Affymetrix GeneChip Mouse Gene 1.0-ST array (Affymetrix, Santa Clara, CA) was used to define gene expression profiles from the samples. Synthesis and labelling of cDNA targets, hybridization and scanning of GeneChips were carried out by the Microarray Core Facility at the City of Hope. Due to the limited starting material, we used a modified two-cycle amplification protocol. Briefly, cRNA was generated using 10ng total RNA according to the manufacturer's protocol by using Affymetrix's GeneChip Whole Transcript Sense Target Labeling Assay in the first cycle of amplification. The cRNA was subjected to RiboMinus kit (Invitrogen, Carlsbad, CA) to remove rRNA. The resulted cRNA was used as the template for another round of amplification using the sense target labeling assay kit. Hybridization cocktails containing 5.5 μg of fragmented, end-labeled cDNA were prepared and applied to GeneChip Mouse Gene 1.0 ST arrays. Hybridization was performed for 16 hours, and the arrays were washed and stained with the GeneChip Fluidics Station 450 using FS450_0007 script. Arrays were scanned at 5-μm resolution using the Affymetrix GCS 3000 7G and GeneChip Operating Software v. 1.4 to produce.CEL intensity files.

The complete dataset has been submitted to the gene expression omnibus data (GEO) public database at NCBI, and the accession number is GSE77208.

### Immunofluorescence staining

Frozen brain sections were prepared from naive and tumor-bearing mice. Immediately after harvest, brains were fixed in paraformaldehyde for four hours before storage in 30% sucrose solution. Brains were embedded in O.C.T. (Tissue-Tek) and 10 μm sections were cut with a cryostat (Leica Microsystem Inc., Bannockburn, IL). Prior to immunofluorescence staining, slides were baked at 37°C and permeabilized in methanol for 15 minutes. After an hour block, slides were incubated with Arginase I (1:100, Goat anti-mouse, Santa Cruz Biotechnology) primary antibody for 2 hours. Slides were then washed with PBS three times for 5 minutes and incubated with secondary antibody (Rabbit anti-goat Alexa Fluor 555, 1:100 dilution Life Technologies, Carlsbad, CA) for another hour. Tissue sections were mounted in Vectashield mounting medium containing 4060-diamidino-2-phenylindole (DAPI) (Vector, Burlingame, CA), imaged with AX-70 fluorescent microscopy (Leica Microsystems Inc., Bannockburn, IL) and prepared by Zeiss LSM Image Browser software.

Human tumor samples were obtained from the biospecimen repository at City of Hope. Immunohistochemical stains were performed on 4 μm sections cut from formalin-fixed and paraffin embedded tumor (FFPET) blocks and stained with a rabbit anti-human Arg1 monoclonal antibody (Clone SP156, BD Transduction Laboratories) at 1:1500 dilution following a 30 minute steam retrieval in DEVA buffer. Double immunostains were performed utilizing a mouse anti-human CD163 monoclonal antibody (Clone 10D6, Vector laboratories, used at 1:100 dilution) and developed using secondary antibodies from Biocare medical. Four separate fields were used to quantify Arg1^+^ TAMs (CD163^+^) and neutrophils (identified by segmented nuclei).

### Bone marrow transplantation

To develop chimeric mice, lethally-irradiated recipient mice (two doses of 550 cGy every four hours) were injected via tail vein with 5 × 10^6^ bone marrow (BM) cells freshly collected from donor mice [14]. The donor mice were euthanized and BM cells were aseptically harvested by flushing femurs with Dulbecco's PBS (DPBS) containing 2% fetal bovine serum. The samples are combined, filtered through a 40 μm nylon mesh, centrifuged, and passed through a 25 gauge needle. Recovered cells were resuspended in DPBS at a concentration of 5 × 10^6^ viable nucleated cells per 200 μL. All animals were given autoclaved water with sulfatrim from 2 days prior and up to 2 weeks after transplantation. Six to eight weeks after transplantation, mice were implanted with intracranial GL261 gliomas. For MP depletion studies, WT/CD11b-TK^mt-30^ chimeric mice were treated with ganciclovir (GCV, 50 mg/kg, i.p. once every other day) four times prior to tumor implantation and until the tissue was harvested.

### Statistical processing of microarray data

Raw intensity measurements of all probe sets were background corrected, normalized and converted into expression measurements using the Affymetrix’s Expression Console v1.1.1. The Bioconductor “ArrayTools” package was then used to identify the genes differentially expressed between the samples. The corresponding p-values were adjusted using the false discovery rate (FDR) method to control the multiple comparisons of large number of genes. Significant genes were selected with a cut-off of adjusted p<0.01 and log2 ratio of 2. Genes were selected from Arginase pathways and analyzed using Partek Genomic Suite [16, 17].

Statistical comparison in all different experimental conditions was performed with the GraphPad Prism software using two-way analysis of variance (ANOVA) or Student’s t-test. A P value of less than 0.05 was considered significant.

## Results

### Characterization of tumor infiltrating immune cells in GL261 gliomas

**Fig 1:**
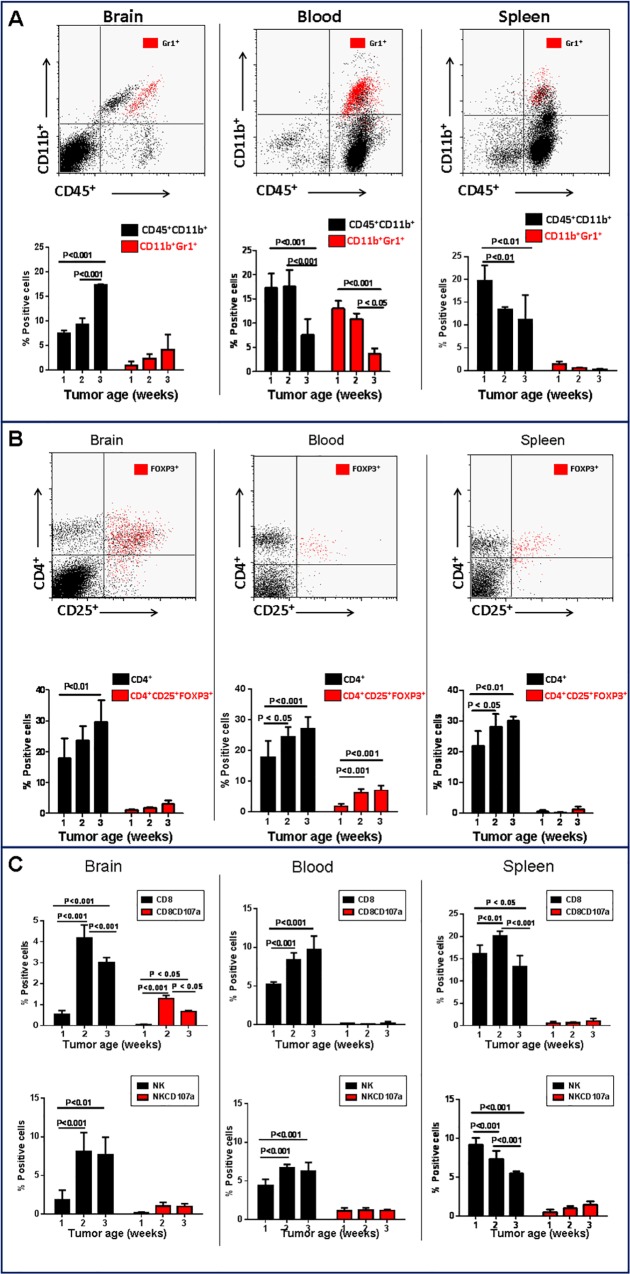
Changes in frequency of immune cell populations during glioma progression. The frequency of different immune subsets was evaluated in brain, blood and spleen during progression of intracranial GL261 gliomas over a three-week period. **A)** Flow cytometry analysis of CD11b^+^CD45^+^ myeloid cells and CD11b^+^Gr1^+^ (marker of myeloid derived suppressor cells). B) Flow cytometry analysis of CD4^+^CD25^+^Foxp3^+^ and **C)** CD8^+^ T and NK cell. Data is representative of two separate experiments. n = 3 mice/group ± SD.

To evaluate the influence of tumor progression on immune cell recruitment, i.c. GL261 gliomas were harvested at different time points after implantation and the proportion of inflammatory cells were quantified by flow cytometry (Fig 1). As predicted, the frequency of tumor-infiltrating cells exhibiting MDSC phenotype (i.e. CD11b^+^Gr1^+^) increased with tumor progression (Fig 1A). Conversely, systemic percentage of CD11b^+^Gr1^+^ cells either decreased (blood) or remained unchanged (spleen) with tumor growth. Further, the frequency of CD4^+^ T lymphocytes expressing the transcription factor FoxP3, a marker of regulatory T cells (Treg), did not significantly change in the tumor microenvironment or in the spleen, but modesty increased in the blood of tumor-bearing mice (Fig 1B). Glioma progression was also associated with an increase in the frequency of both tumor-infiltrating and circulating NK and CD8^+^ cells, but the expression of the activation marker, CD107a, by these cells remained unchanged (Fig 1C). These findings are consistent with concurrent evolution of an immunocompromised tumor microenvironment coupled with systemic immune suppression associated with GL261 progression.

### Gene expression analysis of myeloid-derived subtypes

We reasoned that tumor progression may affect the phenotypic and functional characteristic of infiltrating myeloid cells. To address this hypothesis, an Affymetrix gene array analysis was performed using flow-sorted naïve and tumor-associated myeloid cells (MG: CD11b^+^/CD45^low^; MP: CD11b^+^/CD45^high^) [11]. A large number of transcripts of genes involved in inflammation were upregulated in TAMs and Gr1^+^ cells (S1 Table). Genes involved in anti-inflammatory responses were also upregulated, including a nine-fold increase in Arg1 expression (Table 1). Interestingly, gene expression profiles of tumor associated MP and MG were similar with a few exceptions like S100A8/9 and MMP9 which were down regulated in tumor MP. These observations suggest that glioma progression was associated with changes in the phenotype and functional characteristics of myeloid-derived cells irrespective of their origin. Because Arg1 was upregulated in both tumor MG and MPs, we next analyzed the expression of other associated genes in this pathway.

**Table 1:** Gene expression in GL261-associated myeloid derived cells.

	Tumor MG vs. Normal MG	Tumor MP vs. Normal MP	Gr-1+ vs. Gr-1-
**Inflammatory Response**			
Arg1	9.352	9.396	5.722
Csf1	1.93	2.056	-1.883
Ido1	1.152	1.194	1.131
Ido2	1.259	1.377	1.073
Ifng	-1.13	1.225	-1.209
Igf1	1.285	-1.954	-1.17
Il10	1.044	1.26	1.151
Il1b	6.557	1.968	2.134
Il6	-1.366	1.195	-1.961
Nos2	1.859	1.901	1.746
S100a8	1.535	-17.459	1.37
S100a9	1.811	-20.043	1.558
Stat3	1.323	1.388	1.401
Tgfb1	-1.541	-1.166	1.546
Tnf	2.209	1.471	1.108
**Angiogenic Factors**			
Vegfa	1.894	1.67	1.899
Vegfb	1.011	1.013	-1.349
Pdgfa	1.275	1.156	1.087
Fgf1	-1.22	-1.056	-1.069
Fgf2	-1.128	-1.063	1.076
Ang2	-1.065	-1.121	-1.097
**Proteases**			
Mmp9	1.012	-7.306	1.232
Mmp2	1.297	1.819	-1.748
Mmp14	3.028	3.09	2.531

MG: microglia; MP: macrophages

### Arginase expression in TAMs

**Fig 2:**
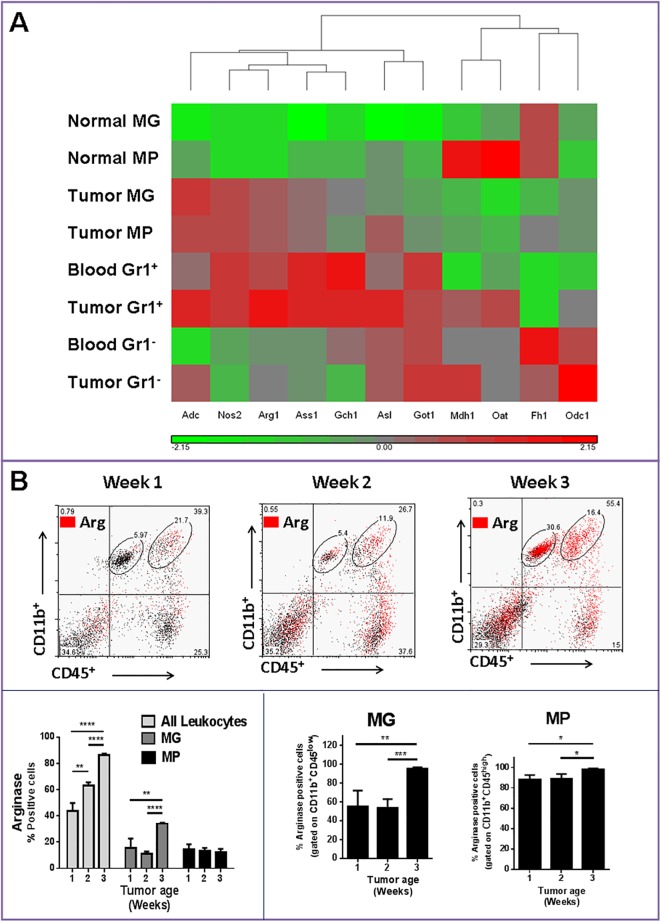
Upregulation of the arginase pathway in tumor infiltrating microglia (MG) and macrophages (MP). **A)** Heat map showing clustering of genes involved in the arginase pathway. All genes shown are upregulated or down regulated more than two fold in tumor-bearing compared to control animals. Grey indicates no change in gene expression, whereas red and green indicate an increase or decrease in expression, respectively, relative to control normal mice. **B)** Flow cytometry analysis of arginase 1 (Arg) expression in MG/MP population during glioma progression. Data is representative of two separate experiments. n = 3–4 mice/group ± SD.

The immunosuppressive function of specific subsets of MDSC depends on multiple mechanisms including the expression of Arg1. This enzyme is responsible for the depletion of the amino acid L-arginine from the microenvironment, which results in suppression of T lymphocytes [3]. Thus, the expression of several genes involved in Arg pathway was analyzed for TAMs (Fig 2A) and Gr1^+^ cells. There was uniform upregulation of this gene cluster (which included Adc, Nos2, Ass1, Gch1, Asl and Got1) in both tumor-associated MP and MG and Gr1^+^ cells (Fig 2A). Blood and tumor-associated Gr1^-^ cells, however, expressed lower levels of these genes. This finding confirms that circulating Gr1^+^ cells are a major contributor to Arg1^+^ cells in this glioma model. However, to assess changes in TAM Arg1 expression with tumor growth, Arg1 expression was also analyzed in myeloid cells at different stages of tumor progression. Using a previously described flow cytometry characterization method (15), we evaluated the expression of Arg in different TAM subpopulations. As expected, tumor growth was associated with enhanced expression of Arg1 in total leukocytes (Fig 2B). While tumor associated CD11b^+^/CD45^high^ (MPs) were a small component of total Arg1^+^ cells (only 10–20%), almost 90% of these cells expressed Arg1. In contrast to MPs, CD11b^+^/CD45^low^ cells (primarily MG) did not express Arg1 initially, but eventually with tumor growth, most MG became Arg1^+^ (Fig 2B). In summary, these findings suggested that secretion of tumor factors resulted in migration of Arg1^+^ cells from the blood and upregulation of Arg1 expression by local CNS MG. To check if migrating Arg1^+^ MPs differentiated into Arg1^+^ MG, we next generated chimeric mice models with differential gene expression in MG and MP cell populations.

### Arg expression in chimeric models

**Fig 3:**
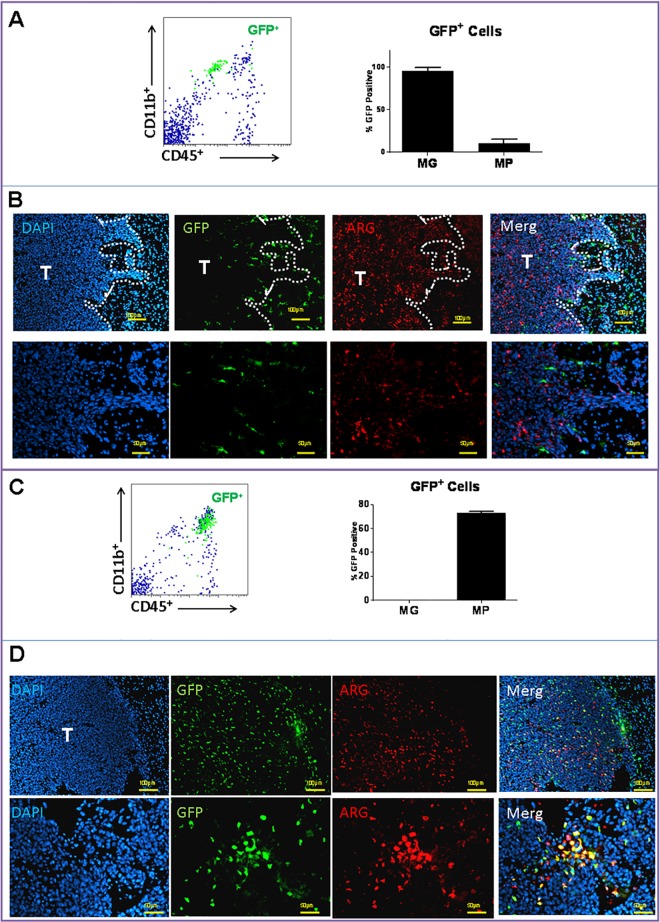
Arginase 1 expression in chimeric mice. Bone marrow cross transplant experiments were performed between donor wild-type (WT) and recipient *CX*_*3*_*CR*_*1*_^*GFP*^ mice. After recovery, mice were implanted with intracranial GL261 tumors and analyzed by **A)** flow cytometry and **B)** histochemistry 10 days later. Tumor-associated microglia (MG), identified as CD11b^+^/CD45^low^ (A) and GFP^+^ cells (B), were located around tumor periphery and were mostly Arg1^-^. In the reverse transplantation experiments, WT recipient mice were transplanted with *CX*_*3*_*CR*_*1*_^*GFP*^ bone marrow prior to tumor implantation. **C)** Glioma-associated macrophages (MP) were identified as CD11b^+^/CD45^high^ and **D)** as GFP^+^ cells and were mostly Arg1^+^. n = 3 mice/group ± SD.

TAMs in gliomas are a heterogeneous cell population that are mainly derived from CNS MG and circulating myeloid cells such as monocytes. In order to evaluate the contribution of each cell type to tumor immunosuppression through Arg1 expression at the initial stages of tumor growth, chimeric mice were generated prior to tumor implantation. The feasibility of this technique was first assessed by cross transplanting BM from WT into *CX*_*3*_*CR*_*1*_^*GFP*^ mice. After recovery, recipient mice were implanted with GL261 tumors and analyzed by flow cytometry and histochemistry 10 days later. Tumor MG (i.e. CD11b^+^/CD45^low^ cells in Fig 3A), mostly remained within the margin of the tumor (Fig 3B, GFP^+^ cells) and were Arg1 negative. Most of the Arg1^+^ cells in this model were in the tumor mass itself (Fig 3B). In the reverse transplantation experiments, where WT recipient mice were transplanted with *CX*_*3*_*CR*_*1*_^*GFP*^ BM, tumor MPs were identified as CD11b^+^/CD45^high^ cells (Fig 3C) and appeared to infiltrate into the tumors (Fig 3D, eGFP^+^ cells). Although many of MPs expressed Arg1, other tumor-infiltrating cells were also Arg1^+^. These cells most likely represented other CD11b^-^Arg1^+^ leukocytes that were also detected by flow cytometry (Fig 2B).

**Fig 4:**
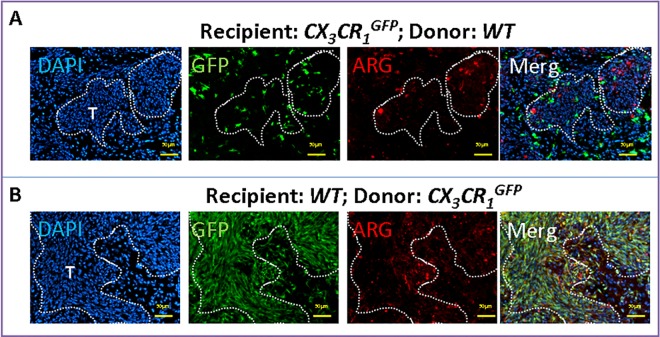
Arginase 1 expression in invasive K-Luc gliomas in chimeric mice. Bone marrow transplant experiments between wild-type (WT) and *CX*_*3*_*CR*_*1*_^*GFP*^ mice were performed. After recovery, mice were implanted with K-Luc tumors and analyzed by histochemistry 10 days later. **A)** In *CX*_*3*_*CR*_*1*_^*GFP*^ recipient mice, microglia were identified as GFP^+^ cells that were Arg1^-^ and surrounded the tumor edge. **B)** In WT recipient mice, GFP^+^ macrophages were mostly Arg1^+^ and infiltrated into the tumor mass.

Expression of Arg by TAMs was also confirmed in the invasive K-Luc glioma model (Fig 4). Here, MG were located at the tumor edge and did not express high levels of Arg1 (Fig 4A). Most of the Arg1 expression was seen in infiltrating MPs (Fig 4B), which were more prevalent in this model than the GL261 gliomas (Fig 3D).

### Effect of MP depletion on MG Arg expression

**Fig 5:**
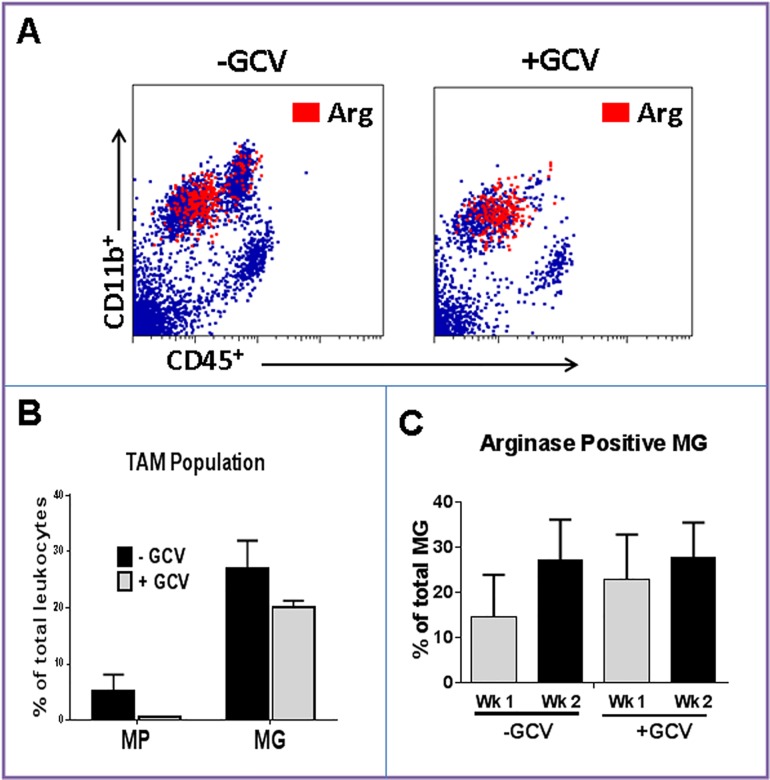
Effect of MP depletion on MG arginase 1 expression. Mice were transplanted with CD11b-TK^mt-30^ bone marrow to generate chimeric mice. After recovery, they were treated with either ganciclovir (+GCV) or PBS (-GCV) and then implanted with intracranial GL261 gliomas. Two weeks later, brains were analyzed by cytometry. **A)** Representative dot plots and **B)** quantification of tumor-associated macrophages (TAM) demonstrating depletion of macrophages (MP, CD11b^+^/CD45^high^) in GCV-treated mice. **C)** Flow cytometry analysis of arginase 1 (Arg) expression demonstrating expression of Arg by tumor-associated microglia (MG) after macrophage (MP) depletion. Data is representative of two separate experiments. n = 3 mice/group ± SD.

To evaluate if the increase in MG Arg1 expression in larger three-week tumors (Fig 2B) was due to differentiation of circulating Gr1^+^ cells into MG or due to tumor microenvironmental influence on resident MG, chimeric models were generated but instead of *CX*_*3*_*CR*_*1*_^*GFP*^, CD11b-TK^mt-30^ mice were used as donor BM. CD11b-TK^mt-30^ mice is a unique mouse model in which the mutant herpes simplex virus type 1 (HSV-1) *TK*^*mt-30*^ gene is expressed under the control of the CD11b promoter, and therefore, is expressed by MG, MPs and monocytes [18]. Treatment of the transgenic mice with GCV (a thymidine kinase inhibitor) eliminated MPs without significantly altering MG population (Fig 5A and 5B). Similar to WT mice, tumor MG in control animals that had undergone BM transplantation without GCV therapy expressed Arg1 within 1 week of tumor implantation and the proportion of Arg1^+^ MG increased with tumor growth (Fig 5C). Interestingly, when MPs were depleted with GCV therapy, MG continued to express Arg1 (Fig 5C). These findings suggest that phenotypic changes in MG in larger tumors were due to local release of tumor factors and not differentiation of Arg1^+^ MP into MG.

### Arg expression in human tumors

**Fig 6:**
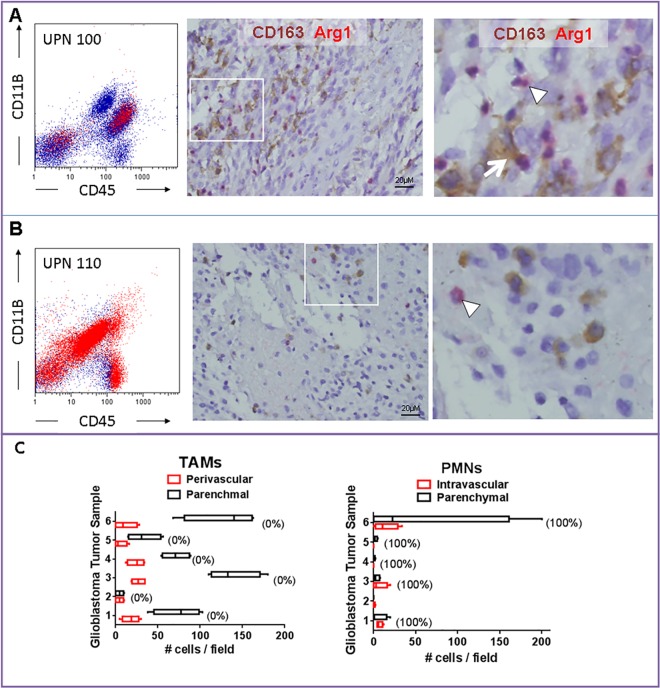
Arginase 1 expression in human malignant glioma samples. Representative tumor tissue from patients with glioblastoma demonstrating heterogeneity of arginase 1 (Arg1) expression by infiltrating inflammatory cells. **A)** In UPN100 (newly diagnosed glioblastoma), both tumor-associated macrophages (TAMs, CD163^+^, arrow) and granulocytes (arrowhead) expressed Arg1. **B)** Most Arg1^+^ cells in UPN110 (recurrent glioblastoma) displayed segmented nuclei characteristic of granulocytes (arrowhead). **C**) Analysis of six additional newly-diagnosed glioblastoma tumor samples revealed that TAMs were more prevalent than neutrophils (PMNs), but nearly all of these cells were Arg1^-^. Numbers in parentheses represent percent Arg1^+^ cells is each cell population.

To confirm Arg1 expression in human tumors, freshly isolated tumor samples were collected from patients undergoing tumor resection and evaluated by flow cytometry and Arg1 histochemistry. The expression of Arg1 was variable among tumor samples. In UPN 100, a patient with newly-diagnosed glioblastoma, flow cytometry demonstrated that most Arg1 expression was in CD11b^+^/CD45^+^ or CD45^-^ tumor-infiltrating leukocytes. (Fig 6A). Tumor histology showed a glioblastoma, characterized by dense tumor areas with interspersed foci of necrosis associate with neutrophils (Fig 6A). Also, numerous interspersed CD163^+^ cells (TAMs) were seen in the tumor mass. Immunohistochemical assessment for Arg1 expression also highlighted numerous tumor-infiltrating leukocytes of predominant neutrophilic lineage (Fig 6A). In UPN 110 (recurrent glioblastoma), Arg1 was detectable in all tumor-infiltrating leukocytes by flow cytometry (Fig 6B). Tumor histology showed recurrent glioblastoma, with less frequent interspersed TAMs and rare Arg1^+^ cells of predominant neutrophilic lineage (Fig 6B). Analysis of six additional newly-diagnosed glioblastoma tissue samples revealed that TAMs were more prevalent than neutrophils, but nearly all of these cells were Arg1^-^. In contrast, every intravascular or intraparenchymal tumor-associated neutrophil was strongly Arg^+^ (Fig 6C). These findings reveal significant heterogeneity in the expression of Arg1 in tumor-infiltrating leukocytes. In addition to TAMs, infiltrating neutrophils appeared to a major contributor to Arg1^+^ cells.

## Discussion

Gliomas are heavily infiltrated by cells of myeloid origin that actively support tumor growth, angiogenesis, invasion and immune evasion [19]. In gliomas, myeloid-derived cells are mostly represented by resident MG and infiltrating MPs. Although these cells are collectively referred to as TAMs, they represent a diverse cell population with potentially different functions. Here, using transgenic mice models we have confirmed that glioma MPs (i.e. CD11b^+^/CD45^high^ cells) mostly arose from circulating Gr1^+^ cells and expressed Arg1 (an M2 marker) shortly after trafficking into tumors. In contrast, tumor MG (i.e. CD11b^+^/CD45^low^ cells) were typically located around tumor edge and expressed Arg1 later during tumor progression. To our knowledge these findings are the first to demonstrate differences in Arg expression between these two cell types in gliomas.

Polarization of TAMs is important to tumor progression. Mounting evidence suggests that glioma-infiltrating MG/MP may promote tumor growth by facilitating immunosuppression of the tumor microenvironment through different mechanisms including Arg1 expression. In small tumors, resident CNS MG accounted for a small fraction of Arg1-expressing cells, however, as tumor progressed, the proportion of Arg1^+^ MG increased. Furthermore, polarization of MG in larger tumors was not dependent on trafficking of circulating myeloid cells, but a function of tumor growth. In the brain, MG can mitigate the tumor-forming capacity of brain tumor initiating cells that contribute to the genesis or recurrence of gliomas, but that capacity is lost when tumors become established [20]. Our findings are consistent with these reports and suggest alterations in MG polarization with tumor growth. Although we did not evaluate specific factors responsible for MG phenotypic conversion in this model, a number of cytokines such as IL-4 and IL-13 can induce Arg1 expression in murine leukocytes. Kohanbash et al showed that GM-CSF secreted by gliomas played a central role in upregulating the expression of IL-1Ra on myeloid cells, which in turn, mediated IL-13-induced production of arginase [21].

Our gene expression analysis, suggested significant similarities between tumor-infiltrating MG and MP in established GL261 gliomas. However, distinct topographic distribution of each cell type within tumors, may suggest different biological functions. In contrast to MPs which were found within hypoxic tumor core, MG were located at the periphery. Role of hypoxia in pro-angiogenic TAM trafficking has been well documented and hypoxia can fine tune the biological activity of M2-like MP subpopulation [22]. Thus, MPs appear to be more involved with angiogenesis and growth of “bulky” component of the gliomas. In contrast, location of MG at the tumor edge is consistent with their role in tumor invasion. In later stages of tumor progression, MG are able to stimulate glioblastoma cell invasion via EGF or MT1-MMP secretion [8, 23]. Expression of Arg1 by these cells at later stages of tumor growth also suggest their role in suppressing the immune response at the tumor edge.

In addition to TAMs, other leukocytes also expressed high levels of Arg1 in both mouse glioma models. These findings were confirmed in human glioma samples where most Arg1^+^ cells were in fact neutrophils and not TAMs. These observations are consistent with other reports demonstrating the presence of circulating suppressive neutrophils in patients with malignant gliomas [24]. Furthermore, the pattern of Arg1 expression by leukocytes may be different in humans than rodents. Although Arg1 is expressed by MP and neutrophils in mice, in humans, Arg1 appears to be expressed only in neutrophils [25]. Therefore, in human glioblastoma, neutorphils may play a more active role in Arg1-mediated local immunosuppression.

Our findings also have clinical implications. Because most Arg1^+^ cells were located within tumor mass, it is possible that a significant contributor to the local tumor immunosuppression can be alleviated through tumor debulking. In fact, previous immunotherapy trials in glioblastoma patients have confirmed best results in patients that undergo a “gross-total” tumor resection. Although it was not examined here, tumor debulking may also abate tumor immunosuppressive signals and reverse the expression of Arg1 by MG surrounding the tumors.

In summary, we have shown significant similarities in gene expression profiles of MG and MPs in an established intracranial glioma model. Although both MG and MP expressed genes that were associated with both M1 and M2 phenotype, polarization of resident CNS MG appeared to occur later during tumor progression. Whether removal of the tumor mass results in reversal of MG polarization will be investigated in future studies.

## Supporting Information

S1 Table Gene expression analysis between Gr1^+^ and Gr1^-^ groups in mouse brain tissue. Partek analysis of gene expression fold change between Gr1^+^ and Gr1^-^ groups. Only genes with fold change difference of at least 2 were included.
(XLS)
